# Mildew-Omics: How Global Analyses Aid the Understanding of Life and Evolution of Powdery Mildews

**DOI:** 10.3389/fpls.2016.00123

**Published:** 2016-02-15

**Authors:** Laurence V. Bindschedler, Ralph Panstruga, Pietro D. Spanu

**Affiliations:** ^1^School of Biological Sciences, Royal Holloway, University of LondonEgham, UK; ^2^Unit of Plant Molecular Cell Biology, Institute for Biology I, RWTH Aachen UniversityAachen, Germany; ^3^Department of Life Sciences, Imperial College LondonLondon, UK

**Keywords:** *Blumeria graminis*, powdery mildews, genomics, transcriptomics, proteomics

## Abstract

The common powdery mildew plant diseases are caused by ascomycete fungi of the order Erysiphales. Their characteristic life style as obligate biotrophs renders functional analyses in these species challenging, mainly because of experimental constraints to genetic manipulation. Global large-scale (“-omics”) approaches are thus particularly valuable and insightful for the characterisation of the life and evolution of powdery mildews. Here we review the knowledge obtained so far from genomic, transcriptomic and proteomic studies in these fungi. We consider current limitations and challenges regarding these surveys and provide an outlook on desired future investigations on the basis of the various –omics technologies.

## Introduction

Infections with fungi that cause powdery mildew disease result in a characteristic white fuzzy patina on the surface of aerial plant organs (primarily leaves and stems). Many plant species can be affected by these pathogenic fungi, displaying characteristic and easily recognizable disease symptoms (Glawe, 2008). All powdery mildews belong to the Erysiphales, an ascomycete order that represents an ancient monophyletic lineage that evolved over 100 million years ago (Takamatsu, 2004; Braun and Cook, 2012). Over this time, they diversified into more than 400 species that are able to colonize nearly 10,000 plant species (Takamatsu, 2004). They are all obligate biotrophic pathogens that establish highly integrated relationships with their hosts. In agriculture, they represent an ever-present threat with a significant impact on the quality and quantity of food plants, forage crops, and ornamentals (Dean et al., 2012).

Large-scale “-omics” techniques such as genomics, transcriptomics, proteomics and metabolomics are known to generate massive and complex data sets (“big data”). In combination, these approaches have the potential to comprehensively dissect a biological system and define how all its components interact dynamically (“systems biology”). Potential integrative insights include the reconstruction of metabolic networks (Droste et al., 2011) and complex signaling pathways (Tieri et al., 2011). Consequently, these methods have been used, individually or in combination, in numerous species and conditions, including different types of fungal phytopathogens (Tan et al., 2009). The handling, integration and interpretation of data from various –omics platforms remains nevertheless a challenging task (Gomez-Cabrero et al., 2014).

In this article, we review the contribution of large-scale –omics studies over the past 15 years to improve our understanding of the fundamental biology of powdery mildew fungi, their evolution, and the relationship with their hosts. In particular, we survey the findings from the first sets of genomic, transcriptomic and proteomic studies and discuss the insights obtained, their significance and inter-relationships.

## Powdery Mildew Genomes

The genomes of several powdery mildew species, *formae speciales*, and isolates have been sequenced, partially assembled, annotated and analyzed (**Table 1**). The best studied to date are those of *Blumeria graminis* f.sp. *hordei*, *B. graminis* f.sp. *tritici* and *Erysiphe necator*, which cause mildews on barley (*Hordeum vulgare*), wheat (*Triticum aestivum*) and grapevine (*Vitis vinifera*), respectively (Spanu et al., 2010; Spanu and Panstruga, 2012; Hacquard et al., 2013; Wicker et al., 2013; Jones et al., 2014; Panstruga and Spanu, 2014). Some limited information on the pea powdery mildew (*E. pisi)* and one *Arabidopsis thaliana*-infecting powdery mildew (*Golovinomyces orontii*) is also available (Spanu et al., 2010). A new significant set of analyses is currently underway in the context of the JGI CSP Project “Comparative Genomics of Powdery Mildews and Associated Plants” (JGI^1^) to expand the taxonomic and pathogenic spectrum: this effort will include the fungi that cause powdery mildews on grapevine (*E. necator*), hops (*Podosphaera macularis*), brassicas (*E. cruciferarum, G. orontii*), tomato [*Pseudooidium* (*neo-*)*lycopersici*], lettuce (*G. cichoracearum* ), pepper (*Leveillula taurica*), cucumber (*P. xanthii*) and strawberry (*P. aphanis*).

**Table 1:** Compilation of powdery mildew omics studies.

Type of –omics study	Powdery mildew species	Impact/key insights	Reference
Genomics			
	*Blumeria graminis* f.sp. *hordei*; *Golovinomyces oontii*, *Erysiphe pisi*	First powdery mildew genomes; genome size; gene number and content; effectors	Spanu et al., 2010
	*Blumeria graminis* f.sp. *tritici*	Evolution of grass powdery mildews	Wicker et al., 2013
	*Blumeria graminis* f.sp. *hordei*	Mosaic haplotype pattern of the barley powdery mildew genome	Hacquard et al., 2013
	*Erysiphe necator*	Copy number variation of *EnCYP51* and its impact on fungicide resistance	Jones et al., 2014
Transcriptomics			
	*Blumeria graminis* f.sp. *hordei*	First powdery mildew ESTs	Thomas et al., 2001
	*Blumeria graminis* f.sp. *hordei*	First time-resolved transcript analysis	Thomas et al., 2002
	*Blumeria graminis* f.sp. *hordei*	First microarray analysis	Both et al., 2005b
	*Blumeria graminis* f.sp. *hordei*	Expression of metabolic pathway genes	Both et al., 2005a
	*Blumeria graminis* f.sp. *hordei*	Haustorial transcriptome (epidermal peels); discovery of the N-terminal effector motif Y/F/WxC	Godfrey et al., 2010
	*Erysiphe necator*	Gene expression during conidiation	Wakefield et al., 2011
	*Erysiphe necator*	Development of microsatellite markers	Frenkel et al., 2012
	*Golovinomyces orontii*	Haustorial transcriptome (isolated haustoria)	Weßling et al., 2012
	*Podosphaera plantaginis*	Single nucleotide polymorphism (SNP) design for metapopulation studies	Tollenaere et al., 2012
	*Blumeria graminis* f.sp. *hordei*	Transcript profile of compatible versus incompatible interaction	Hacquard et al., 2013
	*Erysiphe necator*	Transcriptionally active transposable elements	Jones et al., 2014
Proteomics			
	*Blumeria graminis* f.sp. *hordei*	Asexual spore proteome	Noir et al., 2009
	*Blumeria graminis* f.sp. *hordei*	First comparative proteomic analysis (spores, epiphytic sporulating hyphae and haustoria)	Bindschedler et al., 2009
	*Blumeria graminis* f.sp. *hordei*	Haustorial proteome (isolated haustoria)	Godfrey et al., 2009
	*Blumeria graminis* f.sp. *hordei*	Large-scale proteogenomics; proteome of haustoria and sporulating hyphae	Bindschedler et al., 2011
	*Blumeria graminis* f.sp. *hordei*	Identification of EKA proteins	Amselem et al., 2015b

The initial sequencing efforts yielded some striking and unexpected results. The first surprise was that the genome sizes are much larger than expected. At the time, two sets of ideas influenced the expectation: the average size of genomes from filamentous ascomycetes that had been fully sequenced and assembled was around 40 Mb (e.g., *Neurospora crassa* and *Magnaporthe oryzae*; Galagan et al., 2003; Dean et al., 2005); moreover, the obligate parasitic life-style of the powdery mildew predicated a reduction in genome size and complexity, in line with the trend toward generalized simplification of body, development and genomes seen in many parasites (Poulin and Randhawa, 2015). This forecast turned out to be spectacularly wrong: the genomes of *B. graminis* and *E. necator* species are in fact ∼120–180 Mb, i.e., several times larger than closely related ascomycetes. Comparable findings were observed in some taxonomically unrelated fungi that have similar biotrophic lifestyles, such as the fungi causing rusts (Duplessis et al., 2011) and the mycorrhizal truﬄes (Martin et al., 2010).

In all these cases, the extraordinary expansion in genome size is caused by a massive accumulation of repetitive DNA that is the result of retro-transposon activity throughout the evolution of these fungi (Spanu et al., 2010; Wicker et al., 2013; Jones et al., 2014; Amselem et al., 2015a). There is evidence that these retro-transposons are still active, because several transcripts and proteins encoded by these elements have been identified in powdery mildew transcriptomes and proteomes, respectively (Jones et al., 2014; Amselem et al., 2015b; see also below).

**FIGURE 1:**
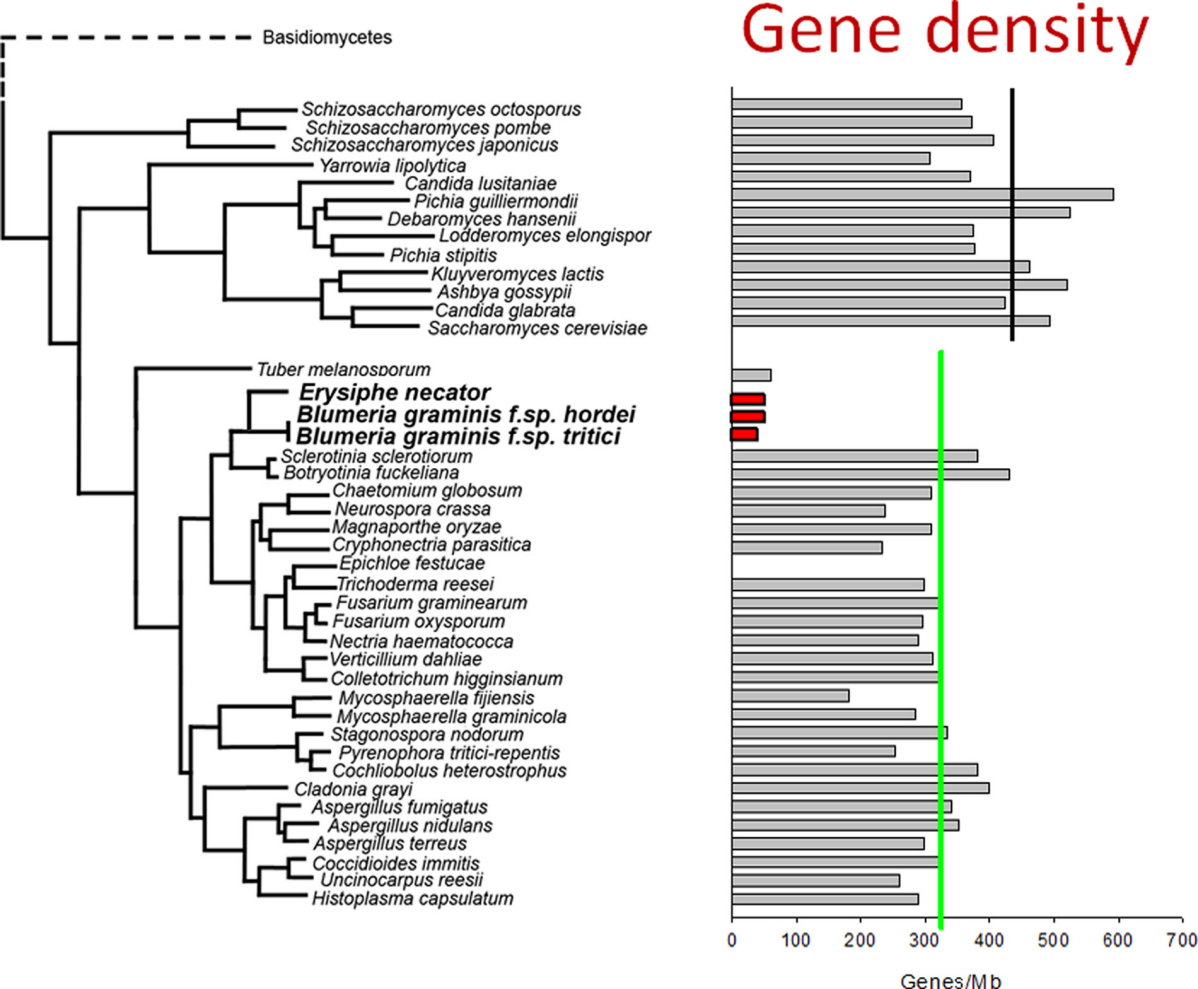
**Low gene density in the genomes of powdery mildew fungi.** The protein-coding gene densities of hemiascomycete and ascomycete genomes were calculated based on published genome sizes and the gene counts of the annotated genomes. The values are plotted as a bar diagram that shows relative taxonomic positions (in a similar way as was used to display relative genome size in a previous study; Spanu et al., 2010). The average gene density of hemiascomycetes (black line) and ascomycetes (green line) are indicated. The gene density in the annotated *Blumeria graminis* genomes (red bars) and of the mycorrhizal truﬄe fungus (*Tuber melanosporum*) are remarkably lower than those of other ascomycetes. This is the result of both increased genome size and loss of some protein–coding genes, as discussed in the text.

The increase in powdery mildew genome size is accompanied by a reduction in the number of protein-coding genes. Around 6,500 protein-coding genes have been identified in *B. graminis* and *E. necator* (Spanu et al., 2010; Wicker et al., 2013; Jones et al., 2014), a number that is considerably lower than in most other fungal phytopathogens (Schmidt and Panstruga, 2011). Overall, these opposing trends result in a marked decrease in gene density compared to taxonomically related fungi (**Figure 1**). This reduction is the result of smaller size of gene families, the near-absence of paralogs, and the elimination of some conserved ascomycete core genes, including the loss of a few metabolic pathways (Spanu et al., 2010; Wicker et al., 2013; Jones et al., 2014). However, genes for most canonical signaling pathways are still present and intact in the *B. graminis* f.sp. *hordei* genome (Kusch et al., 2014). The loss of genes that are otherwise conserved may be attributed to disruption of the loci caused by retro-transposition (Spanu et al., 2010). The absence of a similar set of metabolic pathways in very distantly related plant parasites such as powdery mildews, rust fungi and downy mildew oomycetes (Spanu et al., 2010) is likely to be an indicator of convergent evolution of these obligate pathogens to inhabit a common ecological niche – the live plant cell.

The first comparative analyses of different isolates of both the barley and the wheat powdery mildews demonstrated that extant genomes are essentially mosaics generated over tens of thousands of years by rare sexual recombination events that date back to prior to the domestication of the respective host cereals (Hacquard et al., 2013; Wicker et al., 2013). The maintenance of isolate diversity at the genomic level suggests that there is still much potential for adaptation.

**FIGURE 2:**
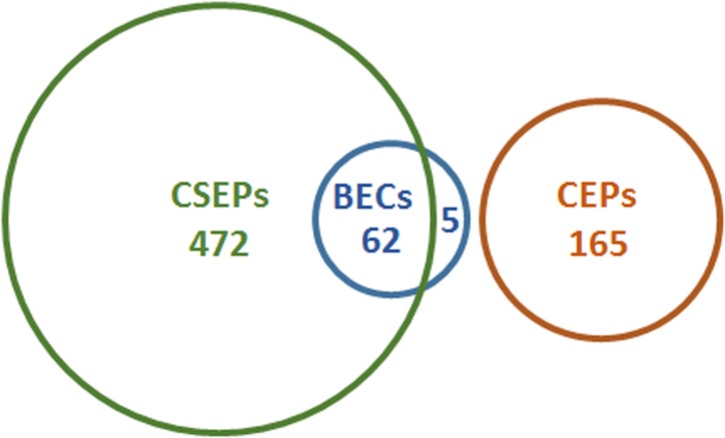
**Candidate effector genes in *B. graminis.*** Several terms are used to name *B. graminis* candidate effectors in the published literature: candidate secreted effector proteins (CSEPs), candidate effector proteins (CEPs), and *Blumeria* effector candidates (BECs). Some of these sets overlap, as shown here for *B. graminis* f.sp. *hordei* in the Euler diagram. CSEPs were originally defined as proteins encoded by bioinformatically annotated genes whose products are predicted to be secreted and that do not have orthologs in non-powdery mildew fungi (found by BLAST searches; Spanu et al., 2010). BECs were defined as proteins identified by protein mass spectrometry that are specifically associated with haustoria and that are predicted to be secreted (Pliego et al., 2013). The five BECs that are not CSEPs include virulence proteins such as BEC1005 and BEC1019, which resemble an endoglycosidase and a metalloprotease, respectively (Pliego et al., 2013; Whigham et al., 2015). Note the high degree of overlap between CSEPs and BECs illustrated by the Euler diagram. Numbers given for these two categories are updated figures from Pedersen et al. (2012) and Bindschedler et al. (2011). CEPs refers to proteins encoded by genes in *B. graminis* f.sp. *tritici* that were identified on the basis of evidence of positive selection (Wicker et al., 2013). While the latter type of analysis has not been carried out for the barley pathogen, it can be assumed that similar numbers exist there because the majority of protein-coding genes has orthologs in both *formae speciales*. The size of the sets as shown in the diagram is proportional to the number of effector candidates identified.

The generation and maintenance of large genomes, full of repetitive DNA, is presumably costly in metabolic terms, and risky in genetic terms, because it can lead to gene disruption by mobile genetic elements. There is evidence that this “cost” is balanced by the advantages posed by active retro-transposition. But what are the terms of this trade-off? The key to explaining this is the existence of an extraordinarily expanded super-family of species- or mildew-specific Candidate Secreted Effector Proteins (CSEPs), on the one hand. Several hundred CSEPs have been identified in the barley and wheat powdery mildew genomes (Pedersen et al., 2012; Wicker et al., 2013; Kusch et al., 2014; **Figure 2**). On the other hand, genes encoding CSEPs are associated with DNA derived from retro-transposons (Pedersen et al., 2012), as are some of the atypical avirulence genes identified in *B. graminis*, which encode non-CSEP proteins (Ridout et al., 2006; Bourras et al., 2015; Amselem et al., 2015b). The concept that effector proteins in filamentous plant pathogens are located in particularly plastic regions of the genomes was first observed in the oomycetes (Raffaele et al., 2010). In the barley powdery mildew fungus, closely related *CSEP* paralogs are physically linked to similar repetitive DNA, suggesting that the increase in *CSEP* numbers in the genome may have been caused by recombination events leading to gene duplications (Pedersen et al., 2012). In fact, genome analysis of *E. necator* revealed that copy number variation is a frequent phenomenon in this powdery mildew species, with ca. 1–5% of the assemblies of five different isolates being subject to this structural genomic adaptation. A striking instance of copy number variation in the *E. necator* genome relates to the *EnCYP51* gene. This gene encodes a cytochrome P450 lanosterol C-14α-demethylase, which is a key enzyme involved in fungal sterol biosynthesis. The respective protein is the target of a class of fungicides termed DMIs (sterol demethylase inhibitors). A single amino acid exchange in CYP51 (Y136F) renders this protein insensitive to DMI fungicides. DNA sequence analysis of 89 *E. necator* isolates showed extensive copy number variation of *EnCYP51*, ranging from one to fourteen copies, which generally correlated with the occurrence of the Y136F mutation. Isolates collected from fungicide-treated vineyards typically were fungicide-resistant and had multiple *CYP51* copies encoding the Y136F variant (Jones et al., 2014). Taken together, the large and highly repetitive powdery mildew genomes may represent ideal substrates for extensive genome plasticity.

Many *B. graminis CSEPs* show significant evidence of positive evolutionary selection pressure, which caused sequence diversification of the encoded proteins (Pedersen et al., 2012; Wicker et al., 2013). Indeed, a novel set of genes encoding candidate effector proteins (CEPs) were identified because of unusually high ratios of non-synonymous to synonymous substitutions that result from positive selection pressure (**Figure 2**). Notably, the CEPs do not have evident canonical secretion signals (signal peptides) and are thus distinct from the CSEPs (Wicker et al., 2013). It remains to be seen whether and how these proteins are actually translocated into the hosts, as is expected of *bona fide* effectors. Poorly characterized non-conventional secretory pathways may need to be invoked here (Ding et al., 2012). Currently, few instances of such non-canonical secretion of phytopathogen-derived proteins have been reported (Ospina-Giraldo et al., 2010; Lowe et al., 2015). Interestingly, the genome of the grapevine powdery mildew pathogen *E. necator* seems to harbor considerably fewer CSEPs than the *B. graminis* genomes (approximately 150 vs. 430–550 CSEPs). Additionally, the 150 *E. necator* effector candidates lack any signs of positive evolutionary selection, which may indicate the current absence of an extensive evolutionary arm’s race between *E. necator* and its plant host, *Vitis vinifera*. This is in accordance with the fact that most cultivated grapevine varieties lack effective powdery mildew resistance genes and are thus susceptible to the disease (Jones et al., 2014). The current challenges in powdery mildew genomics are: broadening the spectrum of species sequenced in this monophyletic group, a deeper analysis of genome variation (including copy number variation) in existing populations, an understanding of the short-term micro-evolutionary potential of these fungi and the establishment of a fully assembled and “finished” reference genome. The first of these challenges (extending the coverage across the order of the Erysiphales) will be met in the next few years through the efforts of a broad international consortium led by Shauna Somerville, Mary Wildermuth and colleagues^2^; one hope is that this information may lead to discoveries and new hypotheses explaining the diversification and host adaptation of these ubiquitous plant pathogenic fungi over their long-term evolution.

Analysis of population genome diversity, particularly in the cereal mildews, could deliver invaluable information about how strains move, distribute across the agronomic spectrum, and change in response to deployment of hosts with new or new combinations of resistance genes. Such a field pathogenomic approach has been highly successful in revealing dynamic changes in wheat yellow rust population structure (Hubbard et al., 2015). Understanding the genetic and genomic responses to fungicide use and comprehending the evolution of resistance to essential pesticides has a great potential to increase our ability to mitigate risk to crop and food security. This challenge is at present unmet in the powdery mildews.

Related to the above, we have very little understanding of the potential for generation of variation through rapid, short-term genetic and/or epigenetic changes in the powdery mildews. A systematic analysis of genome changes in isolated, controlled environments, possibly under diverse selection pressures, will be needed to address this issue.

All these challenges would be greatly facilitated by the availability of fully assembled and finished reference sequences (Thomma et al., 2015). All the powdery mildew genome sequences published to date are highly fragmented. This is due, in large part, to the extremely repetitive character of the genomic DNA, which has made complete assembly impossible with the available technologies. The obligate nature of the organisms themselves also makes it difficult to obtain high amounts of large, intact and pure DNA uncontaminated by host or other associated microorganisms. The availability of new “third generation” sequencing technologies, in particular the direct long-read methodologies (Faino and Thomma, 2014), coupled with very deep “second generation” sequencing and advances in computing and software promise to improve the existing assemblies significantly. It remains to be seen if these enhancements will deliver the full assembly and the complete coverage achieved with other filamentous ascomycetes (Goodwin et al., 2011; Faino et al., 2015). This may be particularly critical because, although the existing genomes have high coverage, the current assemblies are especially poor in the repeat-rich areas. Perversely, these are precisely the areas which appear to harbor a large proportion of the highly interesting genes encoding candidate effectors (CSEPs and CEPs) and EKA family proteins (see below), which are of great relevance to understanding the establishment of the relationship with the host, in particular those modulating host recognition (Bourras et al., 2015). We can thus assume that the effector repertoire of the powdery mildews is even larger than currently known. True completion of finished sequences is therefore of high importance in this respect.

## Powdery Mildew Transcriptomes

A first attempt to study the transcriptome of a powdery mildew pathogen at a larger scale was performed in the pre-genomic era in *B. graminis* f.sp. *hordei* on the basis of expressed sequence tags (ESTs). Using RNA from either ungerminated conidia or conidia germinated on glass plates, two cDNA libraries were generated and used to sequence a random selection of 2,676 clones. This resulted in 4,908 ESTs that represent a total of 1,669 individual sequences. Proteins encoded by these cDNAs were predicted to cover a broad range of different functions (Thomas et al., 2001). Thereafter, serial analysis of gene expression (SAGE) was employed to obtain first insights into the dynamics of gene expression during fungal pathogenesis. SAGE is a method that categorizes cDNAs based on the presence of short oligonucleotide sequences. These sequence tags can then be used to quantify the number of transcripts in a given sample. Thomas et al. (2002) used SAGE to measure the abundance of *B. graminis* f.sp. *hordei* cDNAs in samples from ungerminated conidia, conidia with incipient germ tubes, and germinated conidia with a fully formed appressorium. The authors ended up with 6,336 different tags that were believed to represent unique transcripts. Of these, the 916 tags that occurred at least six times in one of the three samples were used for quantification of cDNA abundance, which revealed different patterns of cDNA accumulation during the early stages of *B. graminis* f.sp. *hordei* pathogenesis. Approximately 20% of the 6,336 tags could be mapped to one of the 1,669 EST-based unigenes previously identified by the same authors (Thomas et al., 2001; see also above), thereby tagging ca. 80% of these unigenes (Thomas et al., 2002).

The next phase in the analysis of powdery mildew transcriptomes was the deployment of cDNA microarrays. Both and co-workers developed a custom-made microarray that harbored 3,327 *B. graminis* f.sp. *hordei* cDNAs representing 2,077 unigenes (Both et al., 2005a,b). These corresponded to cDNAs from conidia, germinating conidia and hyphae (before the onset of conidiation) and included the previously reported EST set (Thomas et al., 2001). The authors utilized the microarray to analyze the transcript profile of *B. graminis* f.sp. *hordei* in the course of barley infection. Eight different RNA samples, derived from heavily inoculated barley plants, were used to synthesize labeled cDNA and probe the microarray. The experiment included four time points prior to host cell penetration (ungerminated conidia; 4, 8 and 15 hpi) and two time points after host cell penetration (3 and 5 dpi) as well as two samples (3 and 5 dpi) from the barley epidermal cell layer after the removal of epiphytic structures, essentially representing haustoria as the main fungal structure. Results of this analysis revealed a global switch in the gene expression pattern between fungal pre- and post-penetration stages, mainly caused by the accumulation of transcripts related to protein biosynthesis (e.g., encoding ribosomal proteins) at later stages of plant colonization. In addition, 51 genes were identified for which the expression profile over time correlated well with the expression of *cap20*, a gene with a well-known virulence function in *Colletotrichum gloeosporioides* (Both et al., 2005b). The same experimental setup further uncovered the coordinated expression of genes that encode enzymes within the same pathways of primary metabolism. Striking examples of this include glycolysis (high transcript levels of the respective genes in mature appressoria and infected epidermis) and lipid metabolism (high transcript levels during fungal germination). These data provided first insights in the type and order of metabolic processes in the course of fungal development and infection (Both et al., 2005a).

To investigate the transcript profile of the grapevine powdery mildew pathogen *E. necator* during development, Wakefield et al. (2011) used the cDNA amplified fragment length polymorphism (cDNA-AFLP) technology, which is a method to fingerprint restriction fragments derived from cDNAs. In this study, the authors put emphasis on later stages of fungal pathogenesis [RNA samples for cDNA synthesis collected prior to conidiation (3 dpi), at conidiophore formation (5 dpi) and during full sporulation (8 dpi)] and also included a sample that represents the formation of sexual ascospores following mating of two opposing mating types (4 weeks post inoculation). The analysis identified 620 cDNA-AFLP fragments that showed differential expression between the four developmental phases under investigation (Wakefield et al., 2011).

A stage-specific transcriptome analysis was conducted on the basis of epidermal peels of heavily *B. graminis* f.sp. *hordei*-infected barley plants from which fungal surface structures were eliminated prior to removal of the epidermal cell layer, as described above. This sample material, which was highly enriched for mature fungal haustoria, was used for cDNA library synthesis and sequencing, which yielded 3,200 unigenes. Among these, 107 candidates for secreted effector protein were identified, which ultimately resulted in the identification of a conserved amino-terminal sequence motif (Y/F/W-x-C) present in the majority of these effector candidates (Godfrey et al., 2010). This sequence motif, whose functional relevance is currently unknown, was later found to be present in many *B. graminis* f.sp. *hordei* CSEPs identified by genome-wide analysis (307 of 491 predicted and analyzed effector proteins; Pedersen et al., 2012).

The advent of next generation sequencing technologies enabled entirely new possibilities for transcriptomic analyses of powdery mildew fungi. For example, the cDNA sequencing of enriched haustorial complexes from *G. orontii*-infected *A. thaliana* plants provided unprecedented insights into the haustorial transcriptome of this powdery mildew pathogen. Sequence analysis of *G. orontii* haustorial cDNAs on the basis of the 454 GS FLX pyrosequencing platform led to the assembly of 7,077 contigs with >5-fold average coverage. Highly represented transcripts encoded proteins involved in protein turnover, detoxification of reactive oxygen species and fungal pathogenesis, including secreted effector candidates. By contrast, transcripts coding for transporter proteins for nutrient uptake were less abundant than expected (Weßling et al., 2012). Transcriptome analysis on the basis of either SOLiD or Illumina short read platforms also assisted annotation of the *B. graminis* and *E. necator* genomes (Spanu et al., 2010; Wicker et al., 2013; Jones et al., 2014) and revealed a multitude of transcripts derived from transposable elements, indicating that these are transcriptionally active (Jones et al., 2014).

Further details of powdery mildew transcriptome dynamics were uncovered in a study that investigated the interaction between *Arabidopsis* and *B. graminis* f.sp. *hordei* by deep Illumina-based RNA-sequencing (Hacquard et al., 2013). Usually, *Arabidopsis* is not a host plant for *B. graminis* f.sp. *hordei*; however, the grass powdery mildew pathogen is able to complete its life cycle on the immuno-compromised *Arabidopsis pen2 pad4 sag101* triple mutant (Lipka et al., 2005). Transcript profiling of early pathogenesis of two *B. graminis* f.sp. *hordei* isolates (a virulent and an avirulent one) on this *Arabidopsis* mutant revealed DNA packaging, nucleosome organization and regulation of chromatin structure as potentially relevant processes around the time of conidium germination (at 6 hpi). During host cell entry (at 12 hpi), the abundance of transcripts related to pathogenesis increased. These also included transcripts coding for CSEPs. In fact, accumulation of *CSEP* transcripts occurred in two successive waves during plant colonization (at 12 and 18–24 hpi). Results of detailed qRT-PCR analyses of a subset of the differentially expressed genes in barley suggest that the *B. graminis* f.sp. *hordei* transcript pattern seen in the non-host plant *Arabidopsis* largely reflects the pattern in the native host, barley. Differences between the transcriptional program of the virulent and avirulent *B. graminis* f.sp. *hordei* isolate concentrated on the 24 h time-point and affected a surprisingly low number of genes (just 76 genes). The majority of these genes (43 of the 76) code for CSEPs, suggesting that the main difference in the fungal expression profile between a compatible and an incompatible interaction resides in the expression of genes encoding effectors (Hacquard et al., 2013).

Finally, next generation-based transcript profiling has been used as a tool to discover markers for population genetic studies. Frenkel et al. (2012) employed 454 GS FLX sequencing of RNA from conidia and mycelium of the grapevine powdery mildew pathogen *E. necator*. This yielded approximately 32,000 sequence contigs that were mined for the presence of microsatellite markers. Of 116 potential markers identified, 31 were tested and detailed, which resulted in 11 microsatellites polymorphic among *E. necator* isolates. Eight of these were then used to analyze the *E. necator* population structure in Europe and North America, which revealed that genetic diversity in eastern USA is much greater than in Europe (Frenkel et al., 2012). A similar approach was applied to study population structure of *P. plantaginis*, the powdery mildew pathogen of *Plantago lanceolata* (plantain), on the archipelago Åland in Finland. Sequencing of RNA extracted from mixed spore material (derived from 16 different isolates) yielded 45,245 sequence contigs which were then mined for single nucleotide polymorphisms (SNPs) for sample genotyping. In the end, a panel of 27 SNP loci was employed for genotyping, which revealed a mixed meta-population of *P. plantaginis*. Additionally, the study disclosed that infection with mixed genotypes on a single host leaf is a common phenomenon within this meta-population (Tollenaere et al., 2012).

Taken together, various attempts have been made in the past 15 years to examine the transcriptome of different powdery mildew fungi (**Table 1**). These comprised studies that either recorded transcript dynamics during fungal pathogenesis (Thomas et al., 2002; Both et al., 2005a,b; Wakefield et al., 2011; Hacquard et al., 2013; Jones et al., 2014) or that focused on a single (pooled) sample (Thomas et al., 2001; Frenkel et al., 2012; Tollenaere et al., 2012) or an enriched infection structure (i.e., haustoria; Godfrey et al., 2010; Weßling et al., 2012). Overall, the studies suffer from a lack of comparability since different host (barley, *Arabidopsis*, *P. lanceolata*, grapevine) and powdery mildew species (*B. graminis* f.sp. *hordei*, *G. orontii*, *P. plantaginis*, and *E. necator*), different experimental techniques (ESTs, SAGE, microarrays, cDNA-AFLP and various next generation sequencing platforms) and in the time-course studies different time-points were used. Moreover, the results have only been partially integrated with the genome (see above) and proteome (see below) data that are now available. Thus, there is still a need for a comprehensive time-resolved transcriptome analysis of a single plant-powdery mildew interaction by deep next generation sequencing that covers the full asexual life cycle from ungerminated conidia to conidiophore formation and sporulation. It would be particularly auspicious if these analyses could be carried out in the context of completely assembled and finished genome sequences. Integration of the data sets would be extremely beneficial in terms of full validation of the gene models in the genome annotation, whilst greatly facilitating the interpretation of global trends in gene expression. In this regard, the above-mentioned JGI CSP Project “Comparative Genomics of Powdery Mildews and Associated Plants” seems especially promising, as it will produce comparative transcriptome data using RNASeq for each sequenced powdery mildew species and its associated host plant at germination, penetration, and proliferation phases of infection.

## Powdery Mildew Proteomes

Large-scale proteome studies of *B. graminis* f.sp. *hordei* were performed on conidia (Bindschedler et al., 2009; Noir et al., 2009), secondary hyphae (Bindschedler et al., 2009, 2011), isolated haustoria (Godfrey et al., 2009) and haustoria in barley epidermis (Bindschedler et al., 2009, 2011; **Table 1**). The resulting peptide information was used during annotation of the *B. graminis* f.sp. *hordei* genome to aid the identification and manual curation of open reading frames (ORFs) and gene models. Based on the ca. 1,500 proteins that had been identified by mass spectrometry (Bindschedler et al., 2011), more than 20% of the 6,500 predicted ORFs were experimentally validated as expressed proteins.

Two-dimensional gel electrophoresis of proteins extracted from ungerminated conidia of *B. graminis* f.sp. *hordei* (Noir et al., 2009) enabled the identification of 123 fungal polypeptides. The main group of proteins belonged to primary metabolism, such as carbohydrate, protein, amino acid, nucleic acid, and lipid/fatty acid metabolism, suggesting that the pathogen is armed for storing compounds, as well as for protein biosynthesis. These findings corroborate those obtained from the survey of transcriptomes based on microarrays (Both et al., 2005a). Additionally, several proteins involved in redox processes and detoxification were found.

The haustorial proteome of purified haustoria (Godfrey et al., 2009), or of infected epidermis following removal of epiphytic hyphae (see above; Bindschedler et al., 2009, 2011), was analyzed by liquid chromatography coupled with nano-electrospray mass spectrometry. In these samples there were many enzymes from primary metabolism. Enzymes associated with alcoholic fermentation, such as pyruvate decarboxylase, were not found, although these proteins are known to be abundant in other fungi (Godfrey et al., 2009). This finding is consistent with the absence of genes encoding these enzymes, as noted when the first *B. graminis* genome was annotated (Spanu et al., 2010). The haustorial proteome is characterized by an overrepresentation of proteins involved in monosaccharide metabolism and stress responses, including heat-shock proteins (Bindschedler et al., 2009, 2011; Godfrey et al., 2009).

Bioinformatic prediction of effectors from pathogenic fungi has been facilitated by the availability of the respective genomes (Schmidt and Panstruga, 2011). However, by definition, effector sequences are diverse and species-specific. Identification often relies on very broad criteria such as small size, predicted protein secretion (i.e., presence of a signal peptide), species-specificity and, possibly, a high cysteine content in the apoplastic effectors (Stergiopoulos and de Wit, 2009).

Large-scale proteomics has contributed to effector discovery through the identification of tissue- or cell-specific *B. graminis* f.sp. *hordei* proteins (Godfrey et al., 2009; Bindschedler et al., 2011). Comparison of the proteomes of haustoria and hyphae revealed that identified haustoria-specific proteins are on average smaller in size than proteins specific to hyphae. Overall, a quarter of the proteins identified only in the haustorial proteome within infected epidermis were not detected in the proteome of sporulating hyphae (Bindschedler et al., 2011). Of these seemingly haustoria-specific proteins, originally 61 and upon refined analysis 67 are predicted to be secreted, i.e. they have a canonical amino-terminal secretion signal and no transmembrane domain. Based on these features they were regarded as *B. graminis* f.sp. *hordei* effector candidates (BECs) expressed in haustoria (**Figure 2**). The large proportion of BECs in the haustorial proteome suggests that they are highly expressed, since proteins in higher abundance (on the basis of more peptides) are usually more easily identified in non-targeted proteome studies. Note that the vast majority of BECs are encoded by *CSEP* genes and are thus identical to matching CSEPs (Pedersen et al., 2012). However, this does not apply to all BECs, since the criterion of the exclusive presence of these proteins in powdery mildews, which was deployed for the classification of the CSEPs, was not used for the assignment of proteins to the BEC category. Two of the BECs that are not CSEPs, are virulence factors necessary for full pathogenic development (Pliego et al., 2013). CSEPs and BECs therefore represent two overlapping and thus in part redundant sets of *Blumeria* effector candidates. (**Figure 2**).

Mining the proteome of purified haustoria led to the identification of more than 200 *B. graminis* f.sp. *hordei* proteins (Godfrey et al., 2009). However, in this study secreted proteins and putative effectors appear under-represented. Conversely, the proteome of infected epidermis allowed the identification of ca. 300 *Blumeria* proteins, including the 67 defined as BECs that are exclusively present in haustoria (Bindschedler et al., 2011). This may be explained by considering that effectors efficiently secreted from haustoria will not accumulate inside them, rendering their detection as intrinsic haustorium peptides a challenging task.

The function in pathogenic development of 50 of the 67 BECs was tested on the basis of host-induced gene silencing (Nowara et al., 2010). Results obtained in this study provided experimental evidence for a virulence role of eight of the effector candidates (Pliego et al., 2013). These included BEC1011 and BEC1054, two RNase-like proteins from CSEP family 21 (CSEP0264 and CSEP0064); BEC1005, a putative glucanase; and BEC1019, a metalloprotease-like protein (Whigham et al., 2015). Mass spectrometry-based proteomics data from a previous study (Bindschedler et al., 2011) were recently re-analyzed to search for the presence of translated products from the large family of retro-transposons associated with the Avrk1 and Avra10 phenotype (EKA family, Ridout et al., 2006), using a novel transposon sequence database that was not available at the time of genome assembly (Spanu et al., 2010). Based on this retrospective examination, several EKA proteins were identified experimentally. Notably, some of the proteins from the retro-transposons associated to Avra10 were found only in haustoria-containing samples (Amselem et al., 2015b).

Taken together, these studies reinforce the notion that *“in planta”* proteomics is an invaluable complement to genomic and transcriptomic studies for the discovery of functional effectors, in particular for biotrophic fungi such as powdery mildews. However, the low biomass of the pathogen in the early stages of infection remains a challenge for such proteomic investigations, which was only partly resolved by the isolation of infected epidermis from barley primary leaves.

An additional bottleneck for the experimental discovery of proteins by mass spectrometry-based proteomics is the dependence on a well-sequenced, well-assembled and ideally also well-annotated genome, since large scale *de novo* peptide sequencing still remains a major technical challenge, despite continuous improvements at the instrument and software level (Ma and Johnson, 2012). Consequently, the lack of ORFs in genome or transcriptome databases conditions that the matching proteins cannot be identified with database-dependent search engines and prediction software. This limitation reinforces the need for further improving genome coverage, assemblies and annotations of powdery mildew fungi.

## Missing And Emerging Mildew-Omes

To our knowledge, there are currently no systematic studies of the powdery mildew metabolome. This could be in part due to the relatively less advanced status of this sub discipline and/or the challenges related to the technologies involved in detection of specific metabolomes of obligate parasites and pathogens growing inside a host. It may also be due to the fact that given the paucity of genes encoding secondary metabolism enzymes in the powdery mildew genomes (Spanu et al., 2010), there has been relatively little impetus to follow this line of investigation, so far. Similarly, systematic large scale approaches to unravel the interaction of powdery mildew proteins with their respective host proteins (interactomics; Collura and Boissy, 2007) are also missing. Up to now, studies mainly focused on individual protein–protein interactions of secreted powdery mildew effector candidates with their potential host targets (Zhang et al., 2012; Schmidt et al., 2014; Ahmed et al., 2015; Pennington et al., in press). A first attempt to investigate such interactions at larger scale resulted in the establishment of a protein–protein interaction network from *G. orontii* haustorial effectors and their respective *Arabidopsis* host proteins. This study revealed convergence of multiple effector proteins on a limited set of host targets (“hubs”) that themselves are highly interconnected with further host proteins and likewise targeted by pathogen effectors from other kingdoms of life (oomycetes and bacteria; Weßling et al., 2014). This work also uncovered a set of host targets that are seemingly specific for *G. orontii* effectors. These plant interactors comprise different types of transcriptional regulators plus a number of proteins with diverse functions (Weßling et al., 2014).

## Outlook For Mildew-Omics

The past 15 years have seen the laying of effective foundations for the large scale survey of genes, transcripts and proteins in the powdery mildew fungi, in spite of the difficulties posed by their obligate biotrophic nature. Advances in methods, technology and analysis software are still required to fill the inevitable gaps that exist. However, as these techniques become more cost-effective, there is an expectation that the missing tesserae of the mosaic will be found and placed in the right order, so that a “big picture” will emerge with greater clarity. These building-blocks are essential for the next challenge: moving the field into a true “systems biology” approach, that is an integration of the information to create realistic modeling of the systems themselves to provide real heuristic value to our investigation. Moreover, because the life style of powdery mildew fungi is inherently intertwined with that of their host, the biggest prize of all will be unraveling the complexity and dynamics of the interactome, for which a start has been recently achieved (Weßling et al., 2014). The site-specific nature of the interaction with the haustoria being in direct contact with host cells poses particular challenges for transcriptomic, proteomic and metabolic studies of these infection structures. Laser microdissection-based enrichment of infection sites, as recently performed in the context of the *Arabidopsis*-powdery mildew interaction (Chandran et al., 2010), is a promising technique to temper this problem.

For all mentioned –omics approaches, the functional validation of identified components is essential. Despite some recent progress in transient transformation protocols (Vela-Corcía et al., 2015), the stable transformation of powdery mildew fungi and thus the targeted generation of knock-out mutants remains elusive. An alternative method for functional assays exploits the phenomenon of host-induced gene silencing (Nowara et al., 2010), which has already been used successfully for the identification of some *Blumeria* effector candidates (Pliego et al., 2013; Whigham et al., 2015). If successful, the integration of insights from –omics studies with the results from functional investigations hold the promise to improve our ability to control these ubiquitous diseases, and mitigating their effect on our food and crop security.

## Author Contributions

LB, RP, and PS jointly worked out the review concept. Primary author of the genome section is PS, primary author of the transcriptome section is RP and primary author of the proteomics section is LB; all other sections were written together. The authors jointly edited the manuscript.

## Conflict of Interest Statement

The authors declare that the research was conducted in the absence of any commercial or financial relationships that could be construed as a potential conflict of interest.

## Footnotes

1 http://jgi.doe.gov/comparative-genomics-of-powdery-mildews/

2 http://jgi.doe.gov/comparative-genomics-of-powdery-mildews/
